# Waking and dreaming consciousness: Neurobiological and functional considerations

**DOI:** 10.1016/j.pneurobio.2012.05.003

**Published:** 2012-07

**Authors:** J.A. Hobson, K.J. Friston

**Affiliations:** a Division of Sleep Medicine, Harvard Medical School, Boston, MA 02215, USA; b The Wellcome Trust Centre for Neuroimaging, Institute of Neurology, University College London, Queen Square, London WC1N 3BG, United Kingdom

**Keywords:** AIM, activation, input-gating and modulation, REM, rapid eye movement, PGO, pontine-geniculate-occipital, LGB, lateral geniculate body, Sleep, Consciousness, Prediction, Free energy, Neuronal coding, Rapid eye movement sleep, Pontine-geniculate-occipital waves, Neuromodulation

## Abstract

This paper presents a theoretical review of rapid eye movement sleep with a special focus on pontine-geniculate-occipital waves and what they might tell us about the functional anatomy of sleep and consciousness. In particular, we review established ideas about the nature and purpose of sleep in terms of protoconsciousness and free energy minimization. By combining these theoretical perspectives, we discover answers to some fundamental questions about sleep: for example, why is homeothermy suspended during sleep? Why is sleep necessary? Why are we not surprised by our dreams? What is the role of synaptic regression in sleep? The imperatives for sleep that emerge also allow us to speculate about the functional role of PGO waves and make some empirical predictions that can, in principle, be tested using recent advances in the modeling of electrophysiological data.

## Highlights

► We present a theoretical review of sleep with a special focus on pontine-geniculate-occipital waves and what they tell us about sleep and consciousness. ► We review the nature and purpose of sleep in terms of protoconsciousness and predictive coding, using the free energy principle. ► By combining these theoretical perspectives, we discover answers to some fundamental questions: such as why is homeothermy suspended during sleep? Why is sleep necessary? Why are we not surprised by our dreams? What is the role of synaptic regression in sleep? ► In brief, we show that the brain can optimize itself during sleep by minimizing the statistical complexity of its model of the waking world. ► The implicit optimization processes are remarkably consistent with the known neurobiology of sleep and provide testable predictions about its functional anatomy.

## 1 Introduction

This review brings together two important themes in neurobiology; the role of sleep in supporting the neuronal processes that underlie consciousness and current formulations of the Bayesian brain based upon Helmholtz's notion of unconscious inference (Helmholtz, 1866/1962). The resulting synthesis reveals a remarkable convergence between an established theory of sleep phenomenology (Hobson, 2009) and recent formulations of brain function in computational terms (Friston, 2010). Specifically, this synthesis provides answers to some fundamental questions about sleep: for example, why is homeothermy suspended during sleep? Why is sleep necessary for Bayes-optimal perception and learning? Why do we dream? Why are we not surprised by our dreams? Why do our eyes move in paradoxical sleep? Why are pontine-geniculate-occipital (PGO) waves so exuberant in rapid eye movement (REM) sleep? What is the role of synaptic regression in sleep? Why is aminergic neuromodulation so central to sleep processes? And so on. Furthermore, it accounts for some basic neurophysiologic aspects of sleep and makes some specific predictions about the functional anatomy of sleep.

In what follows, we review the nature and purpose of sleep in theoretical terms and compare the ensuing predictions with neurobiological data. This review is based on the notion that the brain constitutes a generative model of its sensorium (Dayan et al., 1995): This model or virtual reality has been considered previously in terms of protoconsciousness (Hobson, 2009). According to the protoconsciousness hypothesis; the brain is genetically equipped with a model that generates a virtual reality during sleep and is entrained by sensory input during waking. The basic idea developed here is that sleep is essential to optimize this generative model for Bayes-optimal learning and inference during wakefulness.

**Fig. 1:**
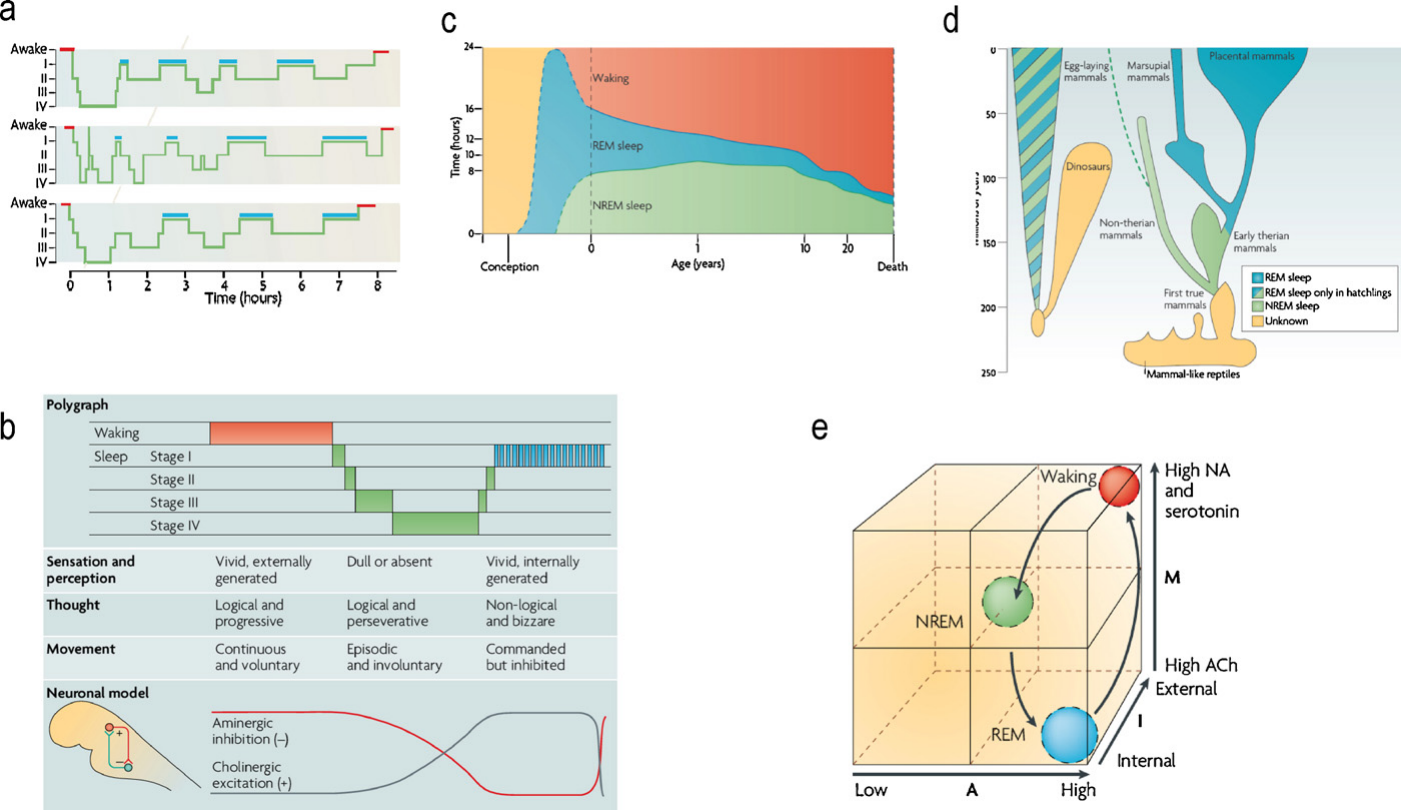
(a) Standard sleep polygraphic measurements. These traces show 90−100 min cycles of rapid eye movement (REM) and non-rapid eye movement (NREM) sleep. The traces show cycles for three subjects, where the blue lines indicate periods of REM sleep. Reports of dreaming are most common from sleep onset stage I (when dreams tend to be fragmentary), late-night stage II (when dreams tend to be thought-like) and REM (when they tend to be long, vivid, hallucinatory and bizarre). Deep phases of sleep (III and IV) occur in the first half of the night, whereas lighter stages (stages I and II) predominate in the second half. (b) The states of waking and sleep. These states have behavioral, polygraphic and psychological correlates that appear to be orchestrated by a control system in the pontine brainstem. In this panel, the neuronal clock that controls these states is depicted as a reciprocal interaction between inhibitory aminergic neurons and excitatory cholinergic neurons: aminergic activity is highest during waking, declines during NREM sleep and is lowest during REM sleep; whereas cholinergic activity shows the reverse pattern. Changes in sleep phase occur whenever the two activity curves cross; these are also the times when major postural shifts occur. The motor immobility during sleep depends on two different mechanisms: disfacilitation during stages I–IV of NREM sleep and inhibition of motor systems during REM sleep. The motor inhibition during REM sleep prevents motor commands from being executed, so that we do not act out our dreams. (c) Human sleep and age. The preponderance of rapid eye movement (REM) sleep in the last trimester of pregnancy and the first year of life decreases progressively as waking time increases. Note that NREM sleep time, like waking time, increases after birth. Despite its early decline, REM sleep continues to occupy approximately 1.5 h per day throughout life. This suggests that its strongest contribution is during neurodevelopment but that it subsequently plays an indispensable role in adulthood. (d) The evolution of REM sleep. Birds and mammals evolved separately after branching off from the ancestral tree. Both birds and mammals are homoeothermic, and both have appreciable cognitive competence. With respect to the enhancement of cognitive skills by REM, it is significant that both birds and mammals are capable of problem solving and both can communicate verbally. (e) AIM model. This panel illustrates normal transitions within the AIM state-space from waking to NREM and then to REM sleep. The *x*-axis represents A (for activation), the *y*-axis represents M (for modulation) and the *z*-axis represents I (for input–output gating). Waking, NREM sleep and REM sleep occupy distinct loci in this space. Waking and REM sleep have high activation but different I and M values. Thus, in REM sleep, the brain is both off-line and chemically differentiated compared with the waking brain. NREM sleep is positioned in the centre of the space because it is intermediate in all quantitative respects between waking and REM sleep.

Adapted from Hobson (2009).

In brief, this optimization is similar to post hoc model selection, applied by scientists after acquiring data. We imagine that sensory data are sampled during wakefulness so that the brain's model can be optimized or learned. Sleep corresponds to the process of post hoc model optimization that prunes redundancy and reduces complexity. The evolutionary imperatives for minimizing complexity may be especially acute for the complex brains of mammals (and birds) that exhibit REM sleep. Crucially, the cycle of model-fitting and pruning emerges through spontaneous and self organized iterations that are mediated by modulatory (aminergic and cholinergic) neurotransmission. We show that the ensuing activation, input-gating and modulation (AIM) model (Hobson, 2009) entails optimization processes that are exactly consistent with the principle of free energy minimization (Friston et al., 2006). See Fig. 1 for a summary of sleep phenomenology and the AIM model.

In Section 2, we consider some of the physiological fundamentals of sleep and emphasize the important role of modulatory neurotransmitters. We then focus on the endogenous excitation of the brain by the PGO waves of REM sleep and consider their relationship to similar activity in waking. Section 4 presents a free energy formulation of sleep in terms of model optimization, based on the empiricism of the preceding sections—our focus here is the optimization of sensorimotor models in the PGO system, during REM sleep. The final section revisits some key empirical issues and concludes with specific predictions about the differences in effective connectivity among components of the PGO system in waking and sleep. This paper establishes the underlying theory and predictions that we hope will be addressed in empirical research over the next few years.

## 2 Sleep and neuromodulation

In this section, we look at the central role of sleep in relation to basic physiology and homoeostasis that underwrites the brain's ability to make conscious and unconscious inferences about the world. In particular, we emphasize the three-way relationship between homeothermy, sleep and consciousness and how these processes depend upon classical modulatory neurotransmitter systems. See Table 1 for a summary. For simplicity, we will ignore differences among species and focus on the neurophysiology of sleep in cats and humans.

It is clear from the experiments of Allan Rechtschaffen that REM sleep is related to the homeostatic control of body temperature, including the temperature of the brain (Rechtschaffen et al., 1989). The coexistence of elaborate corticothalamic circuitry, consciousness and homeothermy in mammals (and birds) is striking and suggests more than a casual correlation. By protecting the very brain structures that support it, homeothermy may be necessary for normal consciousness. Even small variations of brain temperature are devastating to consciousness. Fever destroys our ability to read, much less cogitate. Cold is equally disruptive: one thinks only of how to get warm again.

Neurons that secrete norepinephrine, serotonin and histamine are quiescent in REM (Hilakivi, 1987). Without them animals cannot maintain either waking or active homeothermy. The simplest explanation for the association among REM, homeothermy and consciousness is that REM and temperature control share a common mechanism; namely, aminergic neuromodulation (Docherty and Green, 2010). It is remarkable that REM sleep is the only state of mammalian existence that is not associated with homeothermy (Parmeggiani, 2007). An equally interesting finding is that temperature sensitive neurons in the hypothalamus become temperature insensitive in REM (Parmeggiani, 2003). This finding is consistent with the temperature dependent behavior of Michel Jouvet's pontine cats: these poikilothermic animals stop producing REM if they are cooled and only recover REM when warmed. Furthermore, when humans suppress REM via sustained alcohol ingestion they can develop delirium tremens, in which temperature control is compromised (Hobson, 1999).

These observations indicate that the neural mechanisms underlying REM and homeothermy are linked at a brain stem level and that homeothermy is suspended during REM sleep. What evolutionary pressure could entertain such a risky physiology? Animals do not enter REM sleep if ambient temperature is too high, too low or too unstable (McGinty and Harper, 1976). So why do animals produce REM sleep at all and why does it preclude homeothermy? Clearly, REM sleep must provide an adaptive advantage for those animals that possess it. We will suggest that this advantage rests on optimizing the brain's generative model of its world; and that this optimization necessarily entails suppressing exteroceptive and interoceptive input.

### 2.1 Dreaming and consciousness

REM sleep is the brain state most associated with dreaming (Stickgold et al., 2001). In dreaming, many aspects of primary consciousness are heightened, among them a sense of first person agency, internally generated percepts including movement in fictive space and strong emotions; especially anxiety, elation and anger (Hobson, 2009). Associations, especially remote ones, are enhanced (Spitzer et al., 1991). Conversely, many aspects of secondary consciousness are weakened in sleep: critical judgment, self-reflective awareness, awareness of awareness, orientation, and memory itself are all in abeyance (Hobson, 2009). The most parsimonious way of accounting for these reciprocal changes in phenomenology is to associate them with the known shift in neuromodulatory balance—aminergic neuromodulation is down, while cholinergic and dopaminergic activity is up (Hobson et al., 2000; Solms, 1997). These neuromodulatory mechanisms are an integral part of the protoconsciousness hypothesis: REM sleep (with its dreaming) is fundamental to waking consciousness.

Primary consciousness has been proposed to reflect the machinations of a virtual reality generator that underlies protoconsciousness (Hobson, 2009). Crucially, this virtual reality generator corresponds exactly with the generative models that underlie Helmholtzian perspectives on brain function (Helmholtz, 1866/1962; Barlow, 1974; Gregory, 1980; Dayan et al., 1995; Friston et al., 2006); and, in particular, the free energy principle (Friston, 2010). For both protoconsciousness and Helmholtzian theories, the existence of a predictive mechanism reduces the amount of surprise involved in encounters with external reality (Hobson, 2009; Friston, 2010) and, as we will see later, both call on the same neuromodulatory mechanisms. Hermann von Helmholtz first suggested that the brain must predict the consequences of its sensorimotor activity, in the form of unconscious inference (Helmholtz, 1866/1962). This speaks to the fact that the brain is not a mere reflex organ; it is a synthesizer of sensation, perception and behavior. We suggest that the brain systems responsible for this synthesis are unveiled in REM sleep. One of the most pertinent systems in this regard is the pontine-geniculate-occipital system.

## 3 The PGO system

Since Helmholtz's description of the abnormal percepts produced when abducens palsy patients attempted to move their paralyzed eye (Helmholtz, 1860/1962), we have known that there must be feed-forward information from the eye movement command system in the brain stem to visual centers in the thalamus and cortex. The discovery of the PGO waves during REM sleep in cats provided scientists with a model system for understanding predictive processes in the brain. This section reviews some of the key findings in this area, which are reviewed in terms of predictive coding and free energy minimization in the next section. In brief, the evidence suggests that the PGO network conveys information about eye movements in both waking and REM sleep: however, the enhanced excitability of this system in REM sleep, together with the absence of external visual input, denotes convincing evidence that the brain can generate internal percepts in REM sleep.

### 3.1 A brief history of PGO waves

**Fig. 2:**
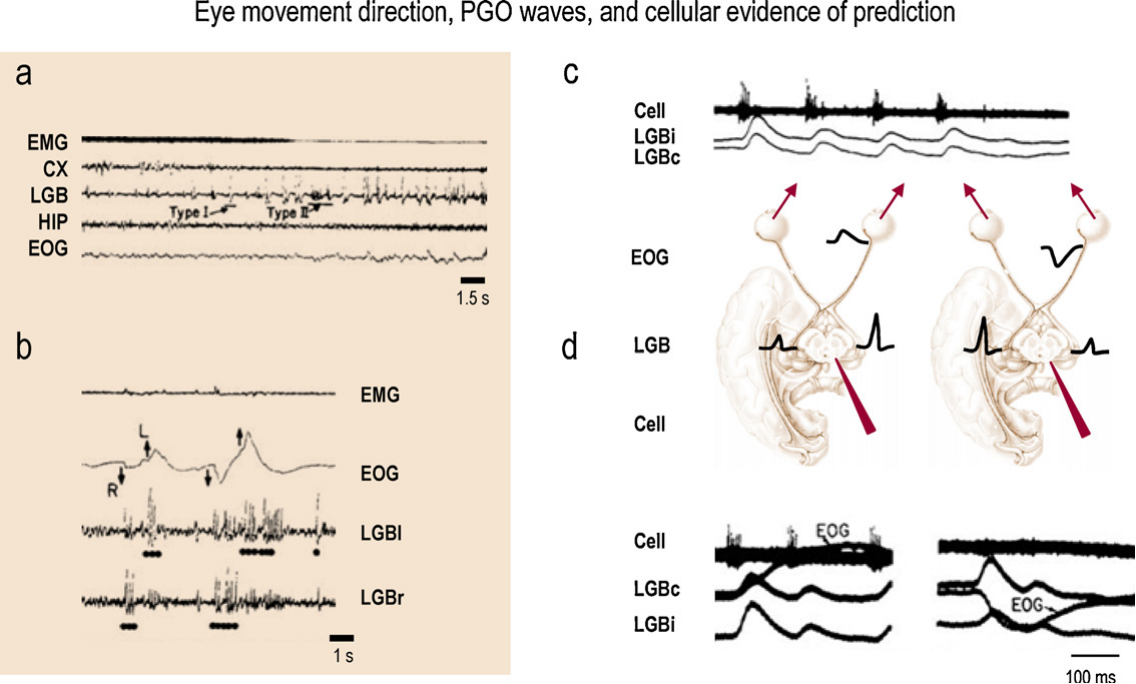
(a) PGO waves and their relation to REM sleep and eye-movements. (A): NREM-REM transition showing PGO waves in LGB (types I and II). During transition periods from NREM to REM sleep, biphasic (PGO) waves in LGB first appear as large single events (type I waves). Waves become clustered with decreasing amplitude (type II waves) as signs of REM sleep become more prominent: atonia (EMG), desynchronization of cortical EEG (Cx), hippocampal- (HIP), and REMs (EOG). (b) Side-to-side alternation of primary waves: Once a REM period is established, predominant PGO wave amplitudes alternate from one geniculate to the other, according to lateral direction of eye movements. When there is rightward movement of the eyes (EOG-R), the corresponding PGO wave cluster is larger in right LGB (dots) than in left. Conversely when there is leftward movement (EOG-L) waves are larger in left LGB (dots). (c) The neuronal firing of a PGO burst cell is shown in the top trace. The PGO waves of the ipsilateral and contralateral geniculate bodies are shown below. It can be seen that the ipsilateral PGO waves are larger in amplitude. In (d) the brain is schematically depicted to reveal eye movement direction. PGO waves form in the two geniculate bodies and PGO burst cell activity in the pons. When the eyes move ipsilaterally (left panel), the cell fires a cluster of spikes prior to the eye movement and prior to the PGO waves. When the eyes move in the opposite direction (right panel), the burst cell is silent and the contralateral PGO wave is twice the amplitude of its ipsilateral counterpart.

PGO waves are large (250 mV) biphasic EEG deflections recorded from the pontine brain stem (P), the lateral geniculate body (nuclei) of the thalamus (G) and the posterolateral cortex (0) of the cat during REM sleep (Fig. 2). We now know that PGO-like activation also characterizes REM sleep in humans and that all sensory systems, not just vision, are affected (Hong et al., 2008). It is significant that they were initially called *activation waves* and likened to visually evoked potentials (Calvet and Bancaud, 1976). However, although PGO waves can arise in response to external stimuli, they are primarily of internal origin (Bizzi and Brooks, 1963; Brooks and Bizzi, 1963).

PGO waves were first observed in 1957 by Mikiten and Hendley in the lateral geniculate body (LGB) of anaesthetized cats but their natural physiology was only appreciated when Michel Jouvet observed them in the REM sleep of cats in 1959. It was Jouvet who named them PGO waves; and from the very start emphasized their possible interpretation as internal signals of obvious significance for dream theory. Were they, Jouvet wondered, the long-sought dream stimuli? If so, might they have the power to unseat Freud's repressed infantile wishes as the instigators of dreams? For a while, PGO waves were thought to propagate from the brain stem to cortex via the thalamus but later work showed the pathways to be independent (Hobson et al., 1969; Laurent et al., 1977). Thus the notion of quasi-visual excitation gradually gave way to the sensorimotor integration paradigm that we emphasize here.

Subsequent work on the PGO wave generation system has not only supported Jouvet's notion about a possible sensory simulation role in relation to dreaming but greatly enriched our understanding of prediction in the brain. In the early days of sleep research in cats, it was not unusual to find that only one of two bipolar twisted wire electrodes was placed accurately enough to reveal geniculate waves. This meant that they were initially regarded as quantifiable REM sleep signatures but were not considered sensorimotor integration signals. It was only when both lateral geniculate body electrodes, in the two sides of the thalamus, recorded bilateral waves that a visible difference in amplitude was apparent: When the eyes moved to the right, the PGO wave in the right LGB was double the amplitude of the wave in the left and vice versa. The same rule applied to the waves in posterolateral cortex. The brain was obviously broadcasting sensory predictions with its motor commands. This is the very essence of predictive processing, as conceived by Helmholtz over 150 years ago (Helmholtz, 1866/1962).

But are PGO waves genuinely internal predictions of upcoming movement or are they merely the consequences of movement? How are PGO waves generated neuronally? What is their timing in relation to ipsiversive and contraversive eye movements? Answers to these crucial questions awaited cellular analysis of the brain stem: As part of an extensive set of studies of pontine brain stem neuronal activity in sleep, Nelson et al. (1983) were able to identify neurons in the parabrachial region of the far-lateral pons that they called PGO burst cells, because they fired in clusters during REM sleep. This firing preceded every high amplitude PGO wave in the LGN and every ipsiversive eye movement. In short, they were able to show that the ipsilateral geniculate PGO wave followed the burst of pontine neuronal firing that anticipated the actual movement of the eye. See Fig. 2.

### 3.2 The neuropharmacology of PGO waves

An appreciation of the PGO system in the context of Lorente de No's vestibulo-ocular reflex (VOR) concept was naturally pursued by the vestibular physiologist Ottavio Pompeiano, who together with Max Valentinuzzi analyzed PGO waves in decerebrate cats. Pompeiano and Valentinuzzi demonstrated that the waves could be potentiated by systemic physostigmine, indicating that acetylcholine might play a role in their generation. That the aminergic neuromodulator, serotonin, might hold PGO waves in inhibitory check was suggested by Raymond Cespuglio, who cooled the midline raphe system with a thermode and thereby released torrents of PGO waves. A similar conclusion was reached by Dana Brooks, whose parasagittal pontine brain stem knife cuts released ipsilateral waves and eye movements. It thus looked as if the PGO system, like REM itself, was cholinergic (on) and aminergic (off).

Confirmation of the cholinergic enhancement and aminergic inhibition of PGO waves and REM sleep rests on studies using drugs that promote or impede neurotransmission in these systems; see (Hobson, 2009). These studies illustrate the sophisticated chemical control of REM by the brain. One surprising observation is the absence of a functional deficit, even after prolonged suppression of REM caused by drugs that inhibit MAO (monoamine oxidase) and thus enhance aminergic inhibition by preventing the enzymatic breakdown of NE and 5-HT. A possible explanation of this paradox is that these drugs do the work of REM by artificially boosting the aminergic system, normally rested in REM.

Another finding of great interest is the uncoupling of PGO Waves and REM caused by the microinjection of cholinergic agonists into the burst cell zone of the far-lateral pons (Silberman et al., 1980; Datta et al., 1992). It is not surprising that this experimental intervention immediately triggers ipsiversive eye movements and large ipsilateral PGO waves, but it is remarkable that no increase in REM is observed until 24 h later. This effect lasts for ten days and cannot be due to the persistent presence of the drug, leading to the hypothesis that cholinergic regulation of REM is in the far-lateral pons, while the trigger zone is more paramedian in a region itself devoid of cholinergic neurons.

### 3.3 PGO waves and prediction

The specificity of the REM sleep-PGO wave association was challenged by Adrian Morrison (Bowker and Morrison, 1976), who observed that unpredicted stimuli in waking were associated with robust PGO waves. Repeated stimuli (which were no longer surprising) failed to elicit these waves. Morrison held that PGO waves reflected the shift in attention needed to analyze an unpredicted (surprising) stimulus: He showed that he could elicit “startle waves” in sleep but they were less likely to habituate when successive stimuli were presented than in waking. Although Morrison did not interpret his results this way, sleep could be said to involve dishabituation of the startle response. Such dishabituation is maximal in REM sleep: put another way, REM sleep is associated with dishabituation of PGO-system activity. This explanation resonates with the physiology of aminergic neurotransmission: demodulation leads to cholinergic potentiation (Hobson, 2009) and hints at a function of surprise or prediction error reduction for REM that will become central in the next section.

Are dreams then our subjective awareness of unbridled startle system activity? If so, why do we rarely experience subjective surprise when dreaming? (Merritt et al., 1994) This question is particularly pertinent to the surprise reduction supposed to be afforded by the PGO system in sleep. Are we made incapable of surprise in dreams because our percepts are the result of top-down predictions in the thalamocortical system? Or is dream memory so impaired that we do not notice novelty? This question cries out for investigation. Our working hypothesis is that dreams ought to be more surprising than they are. Since dream bizarreness reduces to orientation discontinuity and incongruity – and includes cognitive uncertainty, which is not as unpalatable as it would be in waking – we need to explain why we are almost never as startled as we would be if we were exposed to such unpredictable stimuli in waking.

The important observations of Nelson et al. (1983) described above indicate that the ipsilateral geniculate receives a signal that the eyes are about to move before they have actually done so. The information is thus fed forward (and not back) in compliance with Helmholtz and the later notion of efference copy (von Holst and Mittelstaedt, 1950). Nelson et al. also demonstrated that while the excitability of PGO burst cells was about six times greater in REM sleep than in waking, the feed-forward timing relationship between cell firing and eye movement remained unchanged. This meant that predictions about the consequence of eye movements was indeed generated in both states of brain activation and that the generator was much more active when off-line (in REM) than when on-line (in waking).

Subsequent work on the pontine parabrachial region has revealed its significant complexity (Datta and MacLean, 2007) but its functional role does not change with respect to our theoretical treatment. In short, the notion that REM sleep engenders a virtual reality is not only supported but reinforced by neurophysiologic data. The virtual reality system is not only active in REM sleep but it is powerfully amplified. This conclusion provides a possible answer to Leonardo da Vinci's provocative question: *Why does the eye see a thing more clearly in dreams that it does when we are awake?* (Hobson and Wohl, 2005). It seems possible that our visual brains are more strongly excited during sleep than in waking—generating virtual predictions are freed from the task of explaining ambiguous and noisy sensations. Besides its obvious import for a scientific theory of surrealism, it would appear that our nocturnal visions (which we call dreams) are evidence of an epigenetically grounded auto-activation system that is designed to simulate reality. This system is far more robust than subjective experience indicates, because our memory of dreams is evanescent at best. This raises the question of why our dream recall is so impoverished: Perhaps we evolved not to remember our dreams because dreams are the subjective epiphenomena of the nocturnal products of our virtual reality generator and contain no new information.

Until recently, we have had no way of appreciating what function such a mindless activation of the brain might mean. Now, we suggest that REM sleep is a state of the brain that enables essential housekeeping functions, upon which waking consciousness depend. This theory specifies the direct cognitive benefits of REM—just as temperature control is abetted by a state of poikilothermy, so memory function may be enhanced by an amnesic state. The abundant evidence for a functional link between REM sleep and learning in waking is beyond the scope of this review. The interested reader is referred to recent reviews of this intriguing hypothesis (Stickgold and Walker, 2007; Diekelmann and Born, 2010). We will focus on the more basic question—how can the brain learn when a sleep?

### 3.4 Summary

We have seen that there is an intimate relationship between REM sleep, homeothermy and consciousness that rests on neuromodulatory mechanisms. This relationship poses an important question: what is the evolutionary advantage of REM sleep that permits the suspension of homeothermy and that requires the suspension of sensory processing? Important clues lie in the phenomenology of the PGO system that bears all the hallmarks of a predictive system: PGO waves fulfill the requirements of both oculomotor command signals and corollary discharge that allow us to predict the visual consequences of eye movements. Their similarity with the electrophysiological correlates of startle responses again speak to a predictive role: a role that reinforces a view of the brain as a predictive organ that can generate virtual realities (hypotheses) to test against sensory evidence.

The paradox we have to address is that these predictive PGO discharges appear to be augmented during REM sleep, by cholinergic mechanisms, while aminergic neurotransmission gates the sensations that are predicted. How could this paradoxical state of affairs confer evolutionary advantage? We have alluded to the answer in terms of optimizing the brain's model of its world to provide better predictions. In the next section, we review a more formal account of prediction and perception in the brain. This account shows how optimization processes can persist in the absence of sensory input and why this requires the suspension of sensory processing; including the processing of interoceptive signals that elicit homoeothermic responses.

## 4 A free energy formulation of sleep

This section considers sleep in terms of free energy minimization (Friston, 2010). We first review the link between free energy minimization and perception and then consider the difference between perception during waking and sleep. Finally, we consider how these different modes of perception emerge and are maintained in terms of plausible neurobiological mechanisms. The result is a formal description of neuronal dynamics and plasticity during waking and sleep and their quintessential differences. We will see that these differences can be attributed to a single mechanism, whereby internal brain dynamics become sequestered from the sensorium through neuromodulatory gating. The final section revisits the phenomenology of PGO waves in the light of this gating to make some empirical predictions. What follows can be regarded as a free energy formulation of the AIM model (Hobson, 2009).

### 4.1 The free energy principle

The free energy principle is based upon the idea that biological systems resist a natural tendency to disorder (Ashby, 1947) by acting on their environment to minimize something called *surprise*. Surprise (also known as *surprisal* or *self-information*) comes from information theory and quantifies the improbability of sensations, under a model of the world entailed by the brain (Friston, 2010). Because the average of surprise over time is entropy, minimizing surprise enables biological systems to resist the second law of thermodynamics, which says that their entropy or disorder should increase with time. In other words, minimizing surprise allows biological systems to navigate the world in an orderly and predictable way. However, it is impossible to minimize surprise directly. This is where free energy comes in: free energy is always greater than surprise, which means that minimizing free energy implicitly minimizes surprise and endows organisms with a homoeostasis -so that they resist environmental perturbations to the external (e.g., vestibulo-ocular reflexes) and internal milieu (e.g., temperature control) (Ashby, 1947; Bernard, 1974).

The free energy principle formalizes our intuition about what the brain is doing by regarding neuronal activity, neuromodulation and neuronal plasticity as processes that minimize surprise or prediction error. This formulation reveals two important things about sleep: first, it discloses the precise functional or computational role for neuromodulation and, second, it shows that optimization can proceed in the absence of sensory input. This suggests a simple and essential role for sleep, in terms of optimizing the brain's model of its world. In brief, we will see that surprise or free energy corresponds to the difference between bottom-up sensory inputs and top-down predictions of those inputs. This difference is known as prediction error, which means minimizing free energy is basically the same as suppressing prediction error. Prediction errors can be minimized by either changing the sensory input to match predictions or vice versa. These two processes correspond to action and perception, respectively. Crucially, the brain's internal model of the world can only minimize prediction errors if it provides accurate and *parsimonious* explanations of sensory input. It is the optimization of the brain's model per se, in terms of its parsimony or complexity, which we associate with sleep.

Free energy is a very important quantity in this review because it allows us to link ideas about homoeostasis and surprise with statistical concepts like inference and complexity. This means we can contextualize the empirical observations of the previous sections in terms of statistical computations that are implemented by the brain. Box 1 defines free energy mathematically. Put simply, free energy is a quantity that the brain can minimize to reduce surprise. Unlike surprise itself, free energy can be measured by the brain because it is a function of sensory data and internal brain states. These internal states, like neuronal activity and connections strengths, represent the hidden states or causes in the world generating sensations. Changing these representations so that they produce the smallest free energy (prediction error) corresponds to Bayes-optimal perceptual inference (Helmholtz, 1866/1962; Barlow, 1974; Gregory, 1980; Dayan et al., 1995; Friston et al., 2006). Bayesian inference of this sort can be summarized as using sensory information to update *prior beliefs* about the state of the world (that are held before seeing sensory inputs) to produce *posterior beliefs* (that emerge after seeing inputs). In our context, these beliefs correspond to the most likely state of the world that is encoded by neuronal activity and connectivity.

**Fig. 3:**
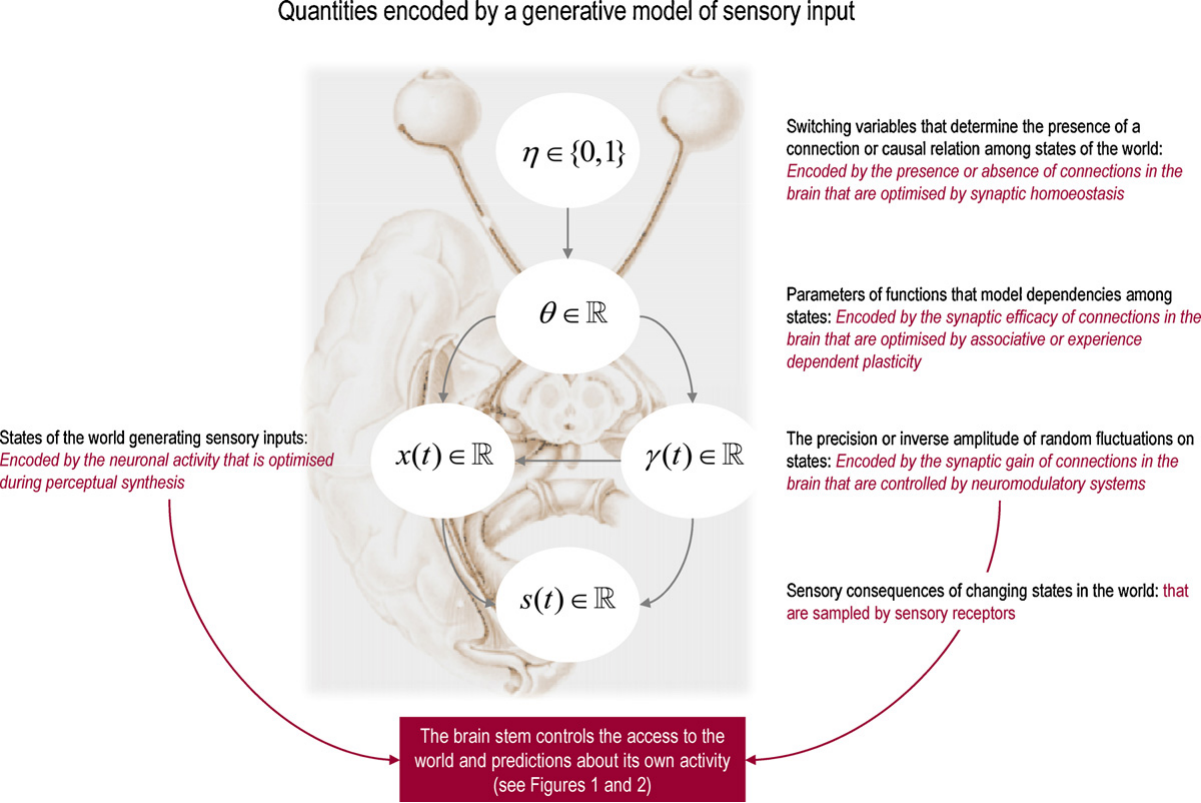
This is a schematic summarizing a generative model of sensory data as a probabilistic graphical model, where the arrows denote statistical dependencies. This is just a formal way of writing down various quantities in a model and how they depend on each other. A generative model can be regarded as a prescription of how to generate a virtual reality and specifies the sorts of quantities required: Some quantities are variables that depend upon time, whereas others specify the causal architecture of the model. These are usually considered to be real valued parameters θ∈ℝ of equations describing the motion of hidden states x(t)∈ℝ and the mapping from hidden states to sensory states s(t)∈ℝ (see Fig. 4). The text in the figure describes the nature of these quantities and how they may be encoded with biophysical or internal brain states. Hidden states correspond to states of the world that generate sensory data; for example, the motion of a visual object and the nature of ambient light that conspire to produce some visual impressions. The switching variables η∈{0,1} at the top can be regarded as priors or constraints on the parameters that determine whether a particular connection or causal dependency among states exists or not.

Clearly, prediction errors rest on the degree to which sensory predictions are violated. These predictions depend on a *generative model* that simulates how various quantities in the world conspire to produce sensory data. It is called a generative model because it can be used to generate sensory data, given the quantities that cause those data (for example a particular object in the field of view). Crucially, these quantities can change quickly (*states* of the world) or slowly (*parameters* describing causal regularities or contingencies that govern changes in states). Furthermore, there is a distinction between the (deterministic) state of the world and the (stochastic) certainty or *precision* with which it is expressed. This means the brain has to represent *states*, *parameters* and *precisions*: Beliefs about states are usually associated with *synaptic activity*, beliefs about parameters correspond to *synaptic efficacy* or connection strengths and beliefs about precision are encoded by *synaptic gain* (see Fig. 3). Having established how the brain may model different quantities in the world, we can now examine the different ways in which free energy can be minimized in waking. We will then show how REM sleep can, perhaps paradoxically, minimize free energy in the absence of sensory input.

### 4.2 Perceptual synthesis and prediction

The brain can only minimize free energy or prediction error with respect to its internal states and action. Here, action corresponds to the motion or configuration of effectors; for example, the activity of alpha motor neurons in the spinal cord, which are active in waking but inhibited in REM sleep (Evarts, 1964; Evarts and Thach, 1969; Pompeiano, 1967). Action redeploys sensory epithelia and therefore determines which sensory inputs are sampled. This means that prediction error can be minimized in two ways, one can either act to ensure that sensory samples conform to predictions or one can change predictions to match sensory samples: These two processes can be regarded as *action* and *perception*, respectively. Intuitively, this process of navigating the world can be thought of as recurrent hypothesis testing (Helmholtz, 1866/1962; Gregory, 1980), by confirming or disconfirming predictions from a virtual reality generator, whose predictions are continuously updated and entrained by prediction errors. To understand this intuitively, imagine feeling your way around a dark room: your careful palpation of surfaces is informed at every point by some internal scene that is constructed inside your head. This virtual reality guides, and is guided by, sensory feedback. Later, we will use the same metaphor to understand vision and saccadic eye movements as visual palpation of the world (O’Regan and Noë, 2001).

When we dream, we create an image of the world entirely within our own brains that is unfettered by sensory feedback. To generate these images or predictions we must have a near infinite storehouse of virtual reality, because our dreams are so richly textured from a perceptual point of view. For example, the dream of a farm by its owner might represent that farm in a myriad different ways, none of which conforms to the actual farm or to any farm ever witnessed in waking. The dreamer is nonetheless satisfied that the farm so fraudulently represented is his, because there are no sensory prediction errors to indicate that his virtual reality is anything but veridical: it is a farm and its condition can be checked. We hypothesize that the reason such polymorphic imagery is not experienced as fictive is because the intrinsic defects in memory are not corrected by sensory input.

#### 4.2.1 The good, the bad and the complex

However, in waking, internal predictions are held to account by sensory input, which has to be predicted accurately without explaining too much detail or sensory noise. In other words, the best predictions are accurate but parsimonious explanations for sensations. Mathematically, this means the predictions should minimize *complexity*. Crucially, the imperative to minimize complexity follows from the imperative to minimize surprise by minimizing free energy. This can be seen by expressing free energy as *complexity* plus *sensory surprise*. See Box 2. Complexity is the difference between posterior and prior beliefs. Complexity reports the degree to which prior beliefs have to be abandoned to predict sensory samples accurately. Mathematically, this corresponds to the degrees of freedom or number of parameters that are called upon to explain data. A good model has low complexity and only updates a small number of parameters to provide a parsimonious explanation for observed data. This will be familiar to many as Occam's razor and is the essence of scientific reductionism: explaining the maximum number of facts with the minimum number of assumptions.

Crucially, prior beliefs about the model's parameters can be regarded as being encoded by the presence or absence of synaptic connections in the brain. This means that complexity can be suppressed by removing redundant synaptic connections and can proceed in the absence of sensory data during sleep (Tononi and Cirelli, 2006). In other words, the brain can continue improving its generative model by reducing its complexity without any new sensory information. Although this may seem counterintuitive, there are many examples of this in the statistics literature, where it is referred to as *model selection* (Friston and Penny, 2011).

Put simply, given a posterior belief (acquired during waking), it is possible to optimize a generative model in a post hoc fashion, after all the data have been observed (during sleep). There are several examples of optimizing models by pruning connection weights in the statistics literature (Williams, 1995; Ponnapalli et al., 1999). These schemes basically remove connections if their strength is insufficient to justify keeping them: see Friston and Penny (2011) for a treatment of this in the context of Bayesian model optimization, which can be considered a generalization of *automatic relevance determination* (MacKay, 1995). We consider post hoc model selection an important metaphor for the optimization of generative models (virtual realities) during sleep. In short, the brain's model corresponds to the myriad of synaptic connections encoding causal regularities in the world. During waking, associative plasticity builds an accurate but overly complex model that is simplified by synaptic pruning during sleep (Tononi and Cirelli, 2006). The nice thing about formulating things in terms of free energy is that one can regard experience-dependent synaptic plasticity during waking as optimizing posterior beliefs, while sleep can be regarded as optimizing prior beliefs through synaptic pruning. Indeed, this separation into two phases of optimization underlies the *wake-sleep algorithm* in machine learning that minimizes free-energy to provide a parsimonious and unsupervised model of data (Hinton et al., 1995).

So why is it important to minimize complexity? Intuitively, a model with low complexity will provide parsimonious explanations for sensory input that will generalize to different situations. It is this ability to generalize that renders a model a veridical representation of the world and enables surprises to be avoided through action. In summary, changes in synaptic efficacy and the expression or regression of synapses underlie the housekeeping and consolidation functions discussed in the previous section. As with all other brain functions, synaptic homeostasis (Gilestro et al., 2009) can be regarded as an optimization process; and can be formulated in a principled and quantifiable way, as minimizing the complexity of generative models employed by the brain. To see what this might look like empirically, we need to consider how the brain minimizes free energy, first when awake and then when asleep.

### 4.3 Predictive coding in the brain

**Fig. 4:**
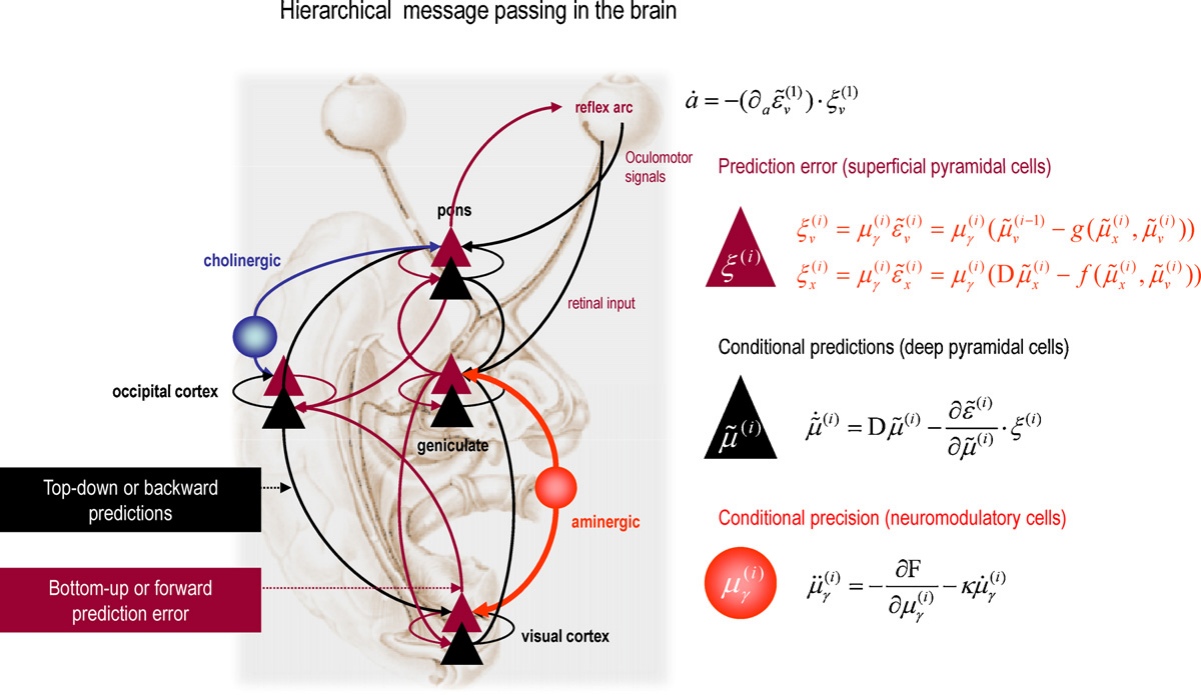
This schematic details a neuronal architecture that optimizes the conditional or posterior expectations about hidden variables in hierarchical models of sensory input of the sort illustrated in Fig. 3. These schemes are based on minimizing the free energy in Box 1 using a gradient descent and can be regarded as a generalization of predictive coding. The particular example here focuses on the PGO system: It shows the putative cells of origin of forward driving connections that convey prediction errors from a lower area to a higher area (red arrows) and nonlinear backward connections (black arrows) that construct predictions (Mumford, 1992; Friston, 2008). These predictions try to explain (cancel) prediction-error in lower levels. In these schemes, the sources of forward and backward connections are superficial and deep pyramidal cells (triangles), respectively, where units representing predictions and prediction error are drawn in black and red, respectively. If we assume that synaptic activity encodes posterior predictions about states, then perceptual inference can be formulated as a gradient descent on free energy: this provides the differential equations shown on the right. Under Gaussian assumptions, these posterior expectations can be expressed compactly in terms of precision weighted prediction-errors: $\left(\xi_{x}^{\left(i\right)},\xi_{v}^{\left(i\right)}\right)$ on the motion of hidden states and causes at the *i*th level of the cortical hierarchy. Here, we have supplemented hidden states with hidden causes that, in hierarchical models, link hierarchical levels. The ensuing equations suggest two neuronal populations that exchange messages; with state-units (black) encoding conditional predictions $\left({\tilde{\mu }}_{x}^{\left(i\right)},{\tilde{\mu }}_{v}^{\left(i\right)}\right)$ and error-units (red) encoding prediction-error. In hierarchical models, error-units receive messages from the state-units in the same level and the level above; whereas state-units are driven by error-units in the same level and the level below. These provide bottom-up messages that drive conditional expectations towards better predictions to explain away prediction-error. Top-down predictions correspond to $g({\tilde{\mu }}_{x}^{\left(i\right)},{\tilde{\mu }}_{v}^{\left(i\right)},\theta )$ and are specified by the generative model, while the dynamics of hidden states are described by the equations of motion $f({\tilde{\mu }}_{x}^{\left(i\right)},{\tilde{\mu }}_{v}^{\left(i\right)},\theta )$. This scheme suggests the only connections that link levels are forward connections conveying prediction errors to state-units and reciprocal backward connections that mediate predictions. Note that the prediction errors that are passed forward are weighted by their conditional precisions, $\left(\mu_{\gamma }^{\left(i\right)}\right)$, that we have associated with the activity of aminergic and cholinergic neuromodulatory systems. Technically, the scheme in this figure corresponds to *generalized* predictive coding because it is a function of generalized variables, which are denoted by a ∼ such that every variable is represented in generalized coordinates of motion: for example, $\tilde{\mu }=(\mu ,\mu^{\prime },\mu^{\prime \prime },\ldots )$. See (Friston, 2008) for further details. In this schematic, occipital cortex sends top-down predictions to visual cortex, which then projects to the lateral geniculate body. However, occipital cortex also sends proprioceptive predictions to the pontine nuclei, which are then passed to the oculomotor system to cause movement through classical reflexes. Predictions from the pontine nuclei are also passed to the lateral geniculate body. These predictions can be thought of as corollary discharge. Every top-down prediction is reciprocated with a bottom-up prediction error to ensure predictions are constrained by sensory information.

There are many ways to model neuronal processing and plasticity as minimizing free energy. The most popular is to regard the brain as performing some form of *predictive coding* (Mumford, 1992; Rao and Ballard, 1999; Friston, 2008). In neurobiological implementations of these schemes (Mumford, 1992), posterior beliefs or predictions about states of the world are represented by the activity of deep pyramidal cells at different levels in sensory cortical hierarchies, while prediction errors are reported by superficial pyramidal cells. Hierarchical signaling between superficial and deep pyramidal cells optimizes predictions by minimizing prediction errors. This process rests upon recurrent forward and backward message passing, where bottom-up prediction errors drive neuronal representations at higher levels of the hierarchy to provide better top-down predictions; thereby suppressing prediction error at lower levels. This means that neuronal activity self-organizes to encode a posterior belief about the states of the world that is optimal in the sense of minimizing prediction error throughout the hierarchy. This corresponds to Bayes-optimal inference about how sensations are caused and is an instance of the Bayesian brain hypothesis (Kersten et al., 2004). Fig. 4 shows an example of this message passing scheme, which we will return to later in the context of sleep.

#### 4.3.1 Predictive coding and action

Crucially, prediction errors at the lowest (sensory) level are the only prediction error that can be suppressed by action. In other words, the only way that action can minimize free energy is by cancelling kinesthetic or proprioceptive prediction errors. This is exactly consistent with classical motor reflex arcs; in which top-down predictions elicit a prediction error in (alpha) motor neurons that contract extrafusal muscle fibers. This elicits reafference from (stretch receptors in) intrafusal muscle spindles until reafference matches descending predictions. An important observation here is that motor command signals descending from the cortex to the spinal-cord (or pontine nuclei) are predictions of a top-down sort that engage classical reflex arcs to ensure predictions are fulfilled. For example, in oculomotor control, descending predictions from the oculomotor system not only inform the visual system the about upcoming visual consequences of eye movement (cf., corollary discharge) but actually cause that movement. The resulting scheme is called *active inference*, in which action is seen as an attempt to minimize surprise by sampling predicted kinesthetic sensations (Friston et al., 2010). This is exactly the functional anatomy implied by the neurophysiology of the PGO system.

Consider how active inference works in the context of the PGO system during saccadic eye movements. Imagine a visual target appears in the peripheral visual field. This will evoke sensory predictions in the lateral geniculate body that will be passed to occipital cortex. These prediction errors will drive deep pyramidal cells in the cortex that encode an orienting saccade to the target. Crucially, the saccade has both visual and proprioceptive consequences that are encoded in the top-down predictions to the LGB and pontine cranial nerve nuclei, respectively. The proprioceptive predictions will elicit proprioceptive prediction errors and an appropriate saccadic eye movement. Coincidentally, top-down predictions to the early visual system will predict that the target will appear in the fovea and, upon completion of the saccade, will be in place to match visual input and thereby suppress visual prediction error. Electrophysiologically, this process would be observed as transient, stimulus-bound prediction errors throughout the PGO system that correspond to startle responses that portend an orienting saccade. We will see later that the same processes are manifest in dreaming; but visual prediction errors are subject to modulatory gating, leaving proprioceptive prediction errors to drive rapid eye movements. In the dream example above, the farmer is not just constructing a farm scene but how he interacts with his virtual reality to generate both visual and oculomotor predictions.

Active inference provides an embodied view of perception, in which the brain actively samples the sensorium using both exteroceptive and proprioceptive predictions. A useful way to think about active inference is that the brain uses amodal representations that are inherently embodied; for example, ‘I am foveating a face’. This representation generates predictions of both the visual and proprioceptive consequences of foveating a face that are fulfilled in an internally consistent way. This is closely related to the notion of seeing as ‘visual palpation’ (O’Regan and Noë, 2001). This example is particularly relevant because many dream faces do not accurately represent the dream characters to which they are assigned (Kahn et al., 2000), anymore than the farm scene corresponds to the dreamer's real farm.

We will see later that this loss of representational accuracy is completely understandable, because there are no visual prediction errors in sleep to constrain representations. A salient example of top-down predictions being under constrained by sensory information is the illusion of a complete object in response to incomplete cues; such as a colleague who suddenly appears under his hat on the rack outside an office (Hobson, 2011). In short, bimodal top-down predictions to visual and proprioceptive streams can be thought of in terms of the distinction between descending motor signals and corollary discharge. We will return to this theme later when considering saccades in rapid eye movement sleep, which we propose are elicited not by (geniculate) *visual* prediction errors but by (pontine) *proprioceptive* prediction errors.

#### 4.3.2 Predictive coding and precision

A key attribute of prediction errors is their precision. Precision determines the influence or potency of prediction errors. Precision can be regarded as an estimate of the signal-to-noise or certainty about predictions. The mathematical form of predictive coding (Fig. 4) suggests that precision is encoded by the postsynaptic gain of cells encoding prediction error. This means that cells with a high gain broadcast a precise prediction error that has more influence on higher levels of processing. This gain or precision has to be optimized in the same way as any other posterior belief and highlights the important role of the classical neuromodulators. For example, we could associate the aminergic projections from the locus coeruleus with an important source of gain control in the early visual pathway and, implicitly, a representation of the precision of sensory signals. Indeed, Aston-Jones et al. (1991) have suggested that norepinephrine increases the signal to noise ratio of neuronal firing to mediate attention, in a way that is exactly consistent with theoretical predictions (Feldman and Friston, 2010). Conversely, one might assign cholinergic projections from the nucleus basalis to a role in representing the precision of prediction errors at higher levels in the cortical hierarchy. Another key neuromodulatory mechanism is the effect of DA-1 agonists on top-down processing (Noudoost and Moore, 2011), which again fits comfortably with precision in predictive coding (Friston et al., 2012). The relative precision of prediction error units at different levels of a hierarchy has a profound influence on the inferential dynamics and resulting predictions (Geisler and Albrecht, 1992; Yu and Dayan, 2005). Precision or neuromodulation plays a central role in our theoretical model of sleep because it has a profound influence on the optimization of synaptic activity, gain and efficacy.

**Fig. 5:**
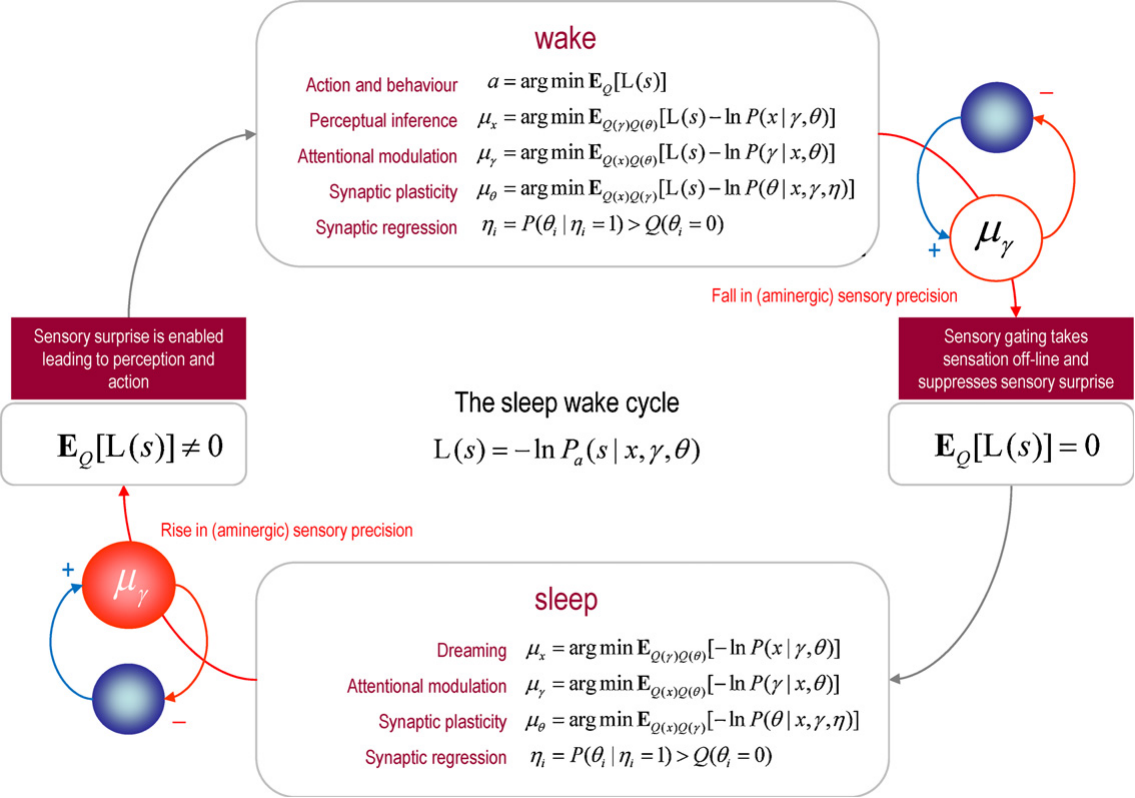
This figure shows how the reciprocal interactions between cholinergic and aminergic systems entrain perceptual processes during the sleep wake cycle. Aminergic modulation plays a critical role in gating or enabling precise sensory information to drive action and perception. When this modulatory gating suppresses sensory information, the brain's optimization processes are no longer informed by precise sensory prediction errors and change quantitatively. The implicit optimization processes during waking (upper panel) and sleep (lower panel) are based on free energy minimization, using the generative models described in the previous two figures. The free energy principle requires all internal brain states encoding posterior beliefs or conditional expectations to minimize free energy. The particular updates shown in this figure are based upon a standard variational Bayesian procedure (Beal, 2003) that allow us unpack the various processes involved. For every quantity in the generative model (see Fig. 3) there is a corresponding update. These updates are computed using conditional expectations denoted by $E_{Q}[\cdot ]$. Here, $\text{L}(s)=-\ln P_{a}(s|x,\gamma ,\theta )$ is sensory surprise and is just sensory prediction error times its precision. Sensory surprise is effectively turned off during sleep because the precision of sensory prediction errors is reduced. Neurobiologically, we assume this is mediated by circadian fluctuations in aminergic neurotransmission (pink circles). The text describes, briefly, the biophysical and neurobiological processes that can be associated with each of the updates. These are largely the same in sleep and wake, with the exception of action that depends exclusively on sensory surprise (that is absent during sleep). We have included priors governing the presence or absence of a particular connection in these updates. Minimizing the free energy of these priors is formally identical to model selection using the Savage-Dickey density ratio: see (Friston and Penny, 2011) for details. Effectively, this removes or prunes redundant model parameters (synaptic connections) to reduce model complexity and minimize free energy. The optimization of the parameters can be seen in the same light, because these effectively minimize the complexity of empirical priors on hidden states.

Mathematically, the advantage of formulating these optimization processes in terms of free energy minimization is that we can see how they depend upon each other. The upper panel of Fig. 5 lists the optimization processes using a standard mathematical procedure called *variational Bayes* (Roweis and Ghahramani, 1999; Beal, 2003): see figure legend for technical details. The key point to take from this list is that the optimization processes map neatly onto key neuronal processes: optimization of action corresponds to motor control and behavior; the optimization of posterior beliefs about states of the world (encoded by synaptic activity) corresponds to perceptual inference; the optimization of posterior beliefs about precision (encoded by synaptic gain) corresponds to attentional processes and salience, while optimizing posterior beliefs about parameters (encoded by synaptic efficacy) underlies perceptual learning and memory.

The processes in Fig. 5 have been expressed as functions of sensory surprise and other probabilities. We have done this to show that optimal action is the only variable that depends exclusively upon sensory surprise. This is very important because if the precision of sensory prediction errors is suppressed by aminergic gating during sleep, then there are no sensory surprises. This means there is no optimal action; however, every other optimization process or update can still proceed in the absence of precise sensory information. We now look more closely at what this means in terms of neuronal activity and plasticity during sleep.

### 4.4 Predictive coding when asleep

Our explanation for the sleep-wake cycle can be summarized as follows: the brain has epigenetically specified beliefs that the precision of its sensory input will show slow (diurnal) fluctuations. Neurobiologically, this suggests the existence of slow fluctuations in modulatory neurotransmitters (that are entrained by sensory cues), which encode sensory precision. These fluctuations basically reflect the prior belief that the amount of precise information in the sensorium will fluctuate; for example, during darkness or when the eyes are closed. As a result, when we go to bed and close our eyes, the postsynaptic gain of sensory prediction error units declines (through reduced aminergic modulation) with a reciprocal increase in the precision of error units in higher cortical areas (mediated by increased cholinergic neurotransmission). This Bayes-optimal sensory gating is consistent with the fact that aminergic projections terminate in superficial layers that are populated by superficial pyramidal cells reporting prediction errors.

The ensuing sleep state is one in which internal predictions are sequestered from sensory constraints. In other words, top-down predictions will fall upon deaf ears (or blind eyes) because the sensory prediction error units have been rendered insensitive through aminergic gating. On the sensory side, this means that the discrepancy between top-down predictions and (the absence of) sensory signals received will not be registered. On the motor side, proprioceptive prediction errors will be silenced and there will be no drive to motor neurons. In short, although the brain can continue to generate successions of sensorimotor predictions (i.e., dreams), they are not disclosed in terms of early sensory cortical responses or motor behavior. However, the brain can continue to optimize itself by changing its synaptic connections.

Mathematically, changing connection strengths in this fashion optimizes the empirical priors on the dynamics of hidden states, while changing the pattern of synaptic connections optimizes the priors on parameters: see Box 2 and (Kiebel and Friston, 2011) for a discussion of synaptic reorganization and free energy minimization. Optimizing connections in this way minimizes the complexity of the model and makes it a better description of causal structure in the waking sensorium. Note that the mechanisms behind perceptual learning or synaptic plasticity and regression are exactly the same as those employed during waking. The only difference is that the neuronal activity encoding posterior beliefs about the current state of the world now comes to represent prior beliefs, which dictate the content of our dreams. In short, sleep provides an opportunity to eliminate the complexity and redundancy accumulated by experience-dependent learning during the day.

Clearly, the simple distinction between sleep and waking in Fig. 5 does not allow us to consider the potentially important distinctions between various sleep stages; particularly between slow wave sleep (SWS) and REM sleep (Datta, 2010). Physiological evidence suggests that both synaptic regression and plasticity contribute to synaptic homoeostasis and do so differently in slow wave and REM sleep: for example, REM sleep is often associated with elevated markers of long-term plasticity and may represent a window of opportunity for hippocampal-dependent consolidation of synaptic connections in distant sites (Ribeiro et al., 2002). Furthermore, long-term potentiation (LTP) is positively correlated with both REM and SWS in the dorsal hippocampus, where synaptic transmission is positively correlated with REM and negatively correlated with SWS (Ravassard et al., 2009). Although the focus of this paper is on REM sleep and dreaming—the theoretical arguments about minimizing complexity can, in principle, be applied to both SWS and REM sleep; however, the underlying synaptic reorganization may show important quantitative differences, in terms of the brain systems involved and the representations that are implicitly refined (e.g., declarative versus procedural).

The lower panel of Fig. 5 summarizes the optimization processes during sleep and their relationship to the same processes during wakefulness. Formally, the only difference is that sensory surprise disappears because it has been inactivated by (aminergic) neuromodulation. Although this account provides a principled rationale for sleep in terms of the optimization of internal models and their priors, it does not account for the emergence of eye movements and the accentuation of dream reports during sleep.

## 5 PGO waves revisited

If sleep rests upon the suppression of sensory prediction error and the implicit suppression of alpha motor neuron drive, why are eye movements observed during REM sleep? Clearly, the scope of model optimization (through synaptic homoeostasis and regression) would be enlarged if it could include the oculomotor system. But why is the oculomotor system engaged during sleep and not other motor systems? One simple answer is that eye movements have no effect on body posture, in contrast to other striatal muscle systems. In other words, eye movements do not call on sensorimotor integration or coordinated locomotion. This means that saccades and other ocular movements can be engaged with impunity and enjoy the benefits of sleep-dependent model optimization, without engaging full active inference.

**Fig. 6:**
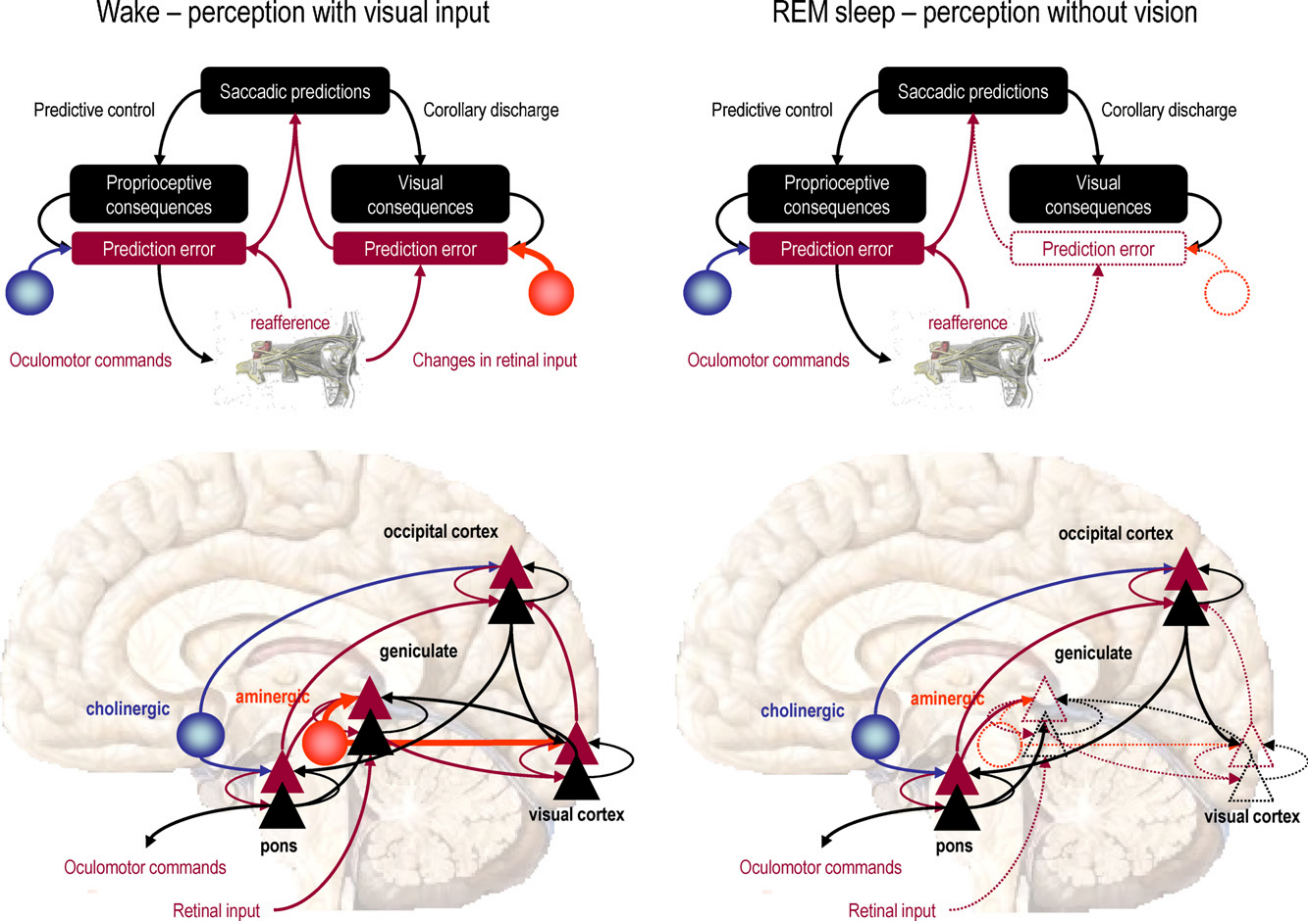
This figure illustrates, schematically, the functional anatomy of visually guided eye movements during waking (left) and REM sleep (right). The upper panels summarize the implicit functional differences in terms of active inference. During wakefulness, top-down predictions about the proprioceptive and exteroceptive consequences of eye movements are sent to pontine and visual centers, respectively. The former elicit eye movements through classical reflex arcs (to suppress proprioceptive prediction error), while the latter anticipate the changes in retinal input. In sleep, there is a selective loss of precision on visual prediction errors. All this means is that the brain thinks its predictions in the visual domain are perfect, because they do not need correcting. This allows for perception without sensation; that is, dreaming. The lower panels show the implicit functional anatomy based on previous figures: this uses a simplified network that comprises the lateral geniculate body (**LGB**), early visual or striate cortex, occipital cortex (that stands in for all high-level cortical areas) and the pontine nuclei controlling eye movements (the cranial nerve nuclei and paramedian pontine reticular formation). Each component of the network is drawn using the principal output cell populations; for example, superficial (dark red) and deep pyramidal cells (black) for forward and backward connections in the cortex Cholinergic (blue) and aminergic (pink) projections control the postsynaptic sensitivity of superficial pyramidal cells that report prediction error and send forward connections. Aminergic projections have been deployed here such that they selectively gate early visual cells in the lateral geniculate body and visual cortex. During sleep, these cells are effectively silenced (denoted by open triangles in the right panel), restricting dynamics to the pontine, geniculate and occipital structures. It is this restriction we associate with the difference between PGO waves in sleep and visually evoked responses associated with orienting saccades during wakefulness. As in Fig. 4, forward connections conveying prediction errors are shown in dark red, while backward connections from state-units that furnish predictions are shown in black. **LC**: Locus Coeruleus and **NBM**: Nucleus Basalis of Meynert.

Fig. 6 illustrates the proposed differences between eye movements in waking and sleep using a schematic simplification of the visual and pontine systems. Many details and neuronal systems have been omitted (such as the frontal eye fields, superior colliculus, etc.) but this simplified network allows us to think about the genesis of eye movements in terms of neuronal dynamics and how they may depend upon the gating of visual information. The left panels picture the state of the brain during waking. Here, aminergic neuromodulation from the locus coeruleus enables retinal input to excite (prediction error) responses in the principal cells of the lateral geniculate body that are then passed forward to striate cortex. As noted above, the ensuing prediction errors engage a recursive hierarchical reorganization of synaptic activity; so that top-down predictions from the extrastriate cortex (e.g., lateral occipital cortex) suppress the prediction errors and, coincidentally, send proprioceptive predictions to the oculomotor system. These predictions then elicit a saccade to foveate the inferred visual object. In short, perceptual inference and subsequent action are driven by precise sensory prediction errors.

In contrast, during sleep, retinal input (even if present) does not have access to the visual cortex and perceptual dynamics rest solely on prior beliefs prescribed by synaptic connections that have been optimized while awake. Given the empirical evidence that PGO waves originate in the pontine system, one might imagine that these circuits rehearse their prior expectations that eye movements will occur sporadically and recurrently. There are several models of this form of itinerancy that, in a biological setting, can be regarded as central pattern generators. We have previously used these to explain perceptual inference on sequences of auditory information (Kiebel et al., 2009) and the generation of sequential motor behavior, like handwriting (Friston et al., 2011).

Sequences of autonomous proprioceptive predictions in the pons will have two consequences: First, they will elicit prediction errors in the cranial nerve nuclei (e.g., the abducens nucleus) that drive eye movements observed during REM sleep. Second, pontine prediction errors will be passed forward to higher (lateral occipital) cortex to drive the perceptual representations that best predict them. In other words, in sleep, proprioceptive prediction errors drive cortical representations to provide a plausible explanation for why the eyes moved, in terms of foveating a salient object. These objects may constitute our visual dream content and are experienced in a way that is free from sensorial constraints (visual prediction errors). From the point of view classical motor control theory, these would constitute corollary discharge [of the sort illustrated in Fig. 6 from the pontine units to the lateral geniculate nucleus].

The perceptual consequences of pontine activity can therefore be seen as playing out in the cortex, engendering conceptual narratives (elicited by forward connections to higher cortical areas) and perceptual representations of an increasingly elemental nature (elicited by backward connections to lower cortical areas) all the way down to the primary visual cortex. This process will continue until predictions encounter prediction error units that have been rendered insensitive through aminergic gating. Clearly, the emergence of PGO waves during REM sleep presupposes that the oculomotor system acquires a selective neuromodulatory boost during periods of REM sleep that we presume is mediated by cholinergic afferents. This fits nicely with the six-fold increase in the excitability of midbrain PGO burst cells in REM sleep, relative to waking (Nelson et al., 1983). In summary, perceptual inference can be regarded as a response to exteroceptive (visual) prediction errors during wakefulness, while the same responses are elicited by proprioceptive (oculomotor) prediction errors during sleep. Put simply, waking percepts are driven by the need to explain unpredicted visual input, while dreaming percepts are driven by the need to explain unpredicted oculomotor input. This interpretation follows directly from the notion of the brain as a generative model of its sensorium that necessarily entails bimodal predictions of exteroceptive and proprioceptive sensations to synthesize its virtual reality (Hobson, 2009).

In terms of electrophysiological responses, the only difference between waking and sleep would be an absence of visually evoked activity in the visual cortex during sleep. This is because the visual cortex is insensitive to top-down or backward afferents and does not receive bottom-up or forward inputs from the gated principal cells in the LGB. This is consistent with the electrophysiological phenomenology of visually evoked startle responses and PGO waves: startle responses and PGO waves are distinguished by the presence and absence of early visual cortical sources. In the context of electrophysiological studies, it is important to note that the cells thought to encode prediction errors (superficial pyramidal cells) are also thought to produce local field potentials and non-invasive electromagnetic signals. Before turning to some specific predictions that follow from this model, we revisit the issue of startle and surprise from Section 3.

### 5.1 Startle versus surprise

Our view of sleep allows us to resolve the apparent paradox between being startled and surprised. Furthermore, it explains why PGO waves show no habituation during sleep, in contrast to visually evoked startle responses during waking. The startle response is elicited by an unpredicted visual stimulus in waking and rests upon precise sensory prediction errors. In sleep, the precision of sensory prediction errors is suppressed and therefore startle responses per se are precluded. In sleep, the brain continues to elaborate saccadic predictions that are fulfilled in terms of eye movements. However, these percepts do not elicit sensory surprise (startle) because they are not constituted by precise sensory prediction errors.

In this context, one can see how startle responses habituate: for example, during the repeated presentation of a peripheral visual stimulus, perceptual learning enables to the brain to predict its occurrence, such that prediction errors are attenuated and cease to elicit an orienting response. This sort of sensory learning has been considered in depth from the point of view of predictive coding, using the mismatch negativity in the auditory domain (Garrido et al., 2009). However, during sleep no such sensory learning is possible because there is no new sensory information and predictions are based purely on empirical priors. In this sense, PGO waves in sleep can be regarded as the neural correlates of percepts that are exempt from the habituation due to sensory learning.

### 5.2 Sleep and thermoregulation

The formulation above provides a simple explanation for the association between sleep and temperature control: if aminergic modulation suppresses the sensitivity of principal cells reporting interoceptive prediction errors, then it will preclude the signaling of thermoreceptors along unmyelinated C-fibers and delta-fibers from various tissues in the body. Recall that temperature sensitive neurons in the hypothalamus become temperature insensitive in REM (Parmeggiani, 2003). In short, the brain will be impervious to fluctuations in temperature and will not respond to suppress thermal prediction errors, resulting in a suspension of homeothermy. So what evolutionary imperatives endorse this risky physiological state? The answer that emerges from our review is that sleep is a natural optimization process that is disclosed by the nightly removal of precise sensory information; in other words, the brain can take itself off-line with impunity, so that synaptic plasticity and homoeostasis (Gilestro et al., 2009) can reduce the complexity it has accrued during wakefulness.

The imperative to reduce complexity during sleep may be greater for the complicated brains of mammals (and birds). The failure to restore complexity to minimal levels would, in principle, mean that experience-dependent learning during the day would not be finessed; leading to a colloquial and context-bound model of the world that becomes increasingly complex and redundant. In statistics, the equivalent pathology is known as ‘over-fitting’ and leads to suboptimal models that fail to generalize beyond the data on which they were trained. In short, taking the brain off-line to prune exuberant associations established during wakefulness may be a necessary price we pay for having a sophisticated cognitive system that can distil complex and subtle associations from sensory samples.

### 5.3 Consciousness and complexity

Although our focus here is on the physiology of the PGO system, it might be useful to speculate on the selective effects of neuromodulation on different aspects of consciousness. The picture that emerges is that perceptual inference and awareness depend on which levels of the cortical hierarchy enjoy the greatest precision (the gain of principal cells reporting prediction error). In this context, the differential effects of various modulatory neurotransmitter systems can be explained by the anatomical specificity of their projection fields. It may be the case that aminergic (norepinephrine, serotonin and histamine) projections preferentially target early sensory structures, while cholinergic projection systems boost the precision of high order sensory and association cortices (including the hippocampus). In this sense, aminergic neuromodulation may control the acuity of sensations, while cholinergic neurotransmission biases towards perceptual synthesis, of the sort associated with primary consciousness.

Finally, it may be that mesocortical and mesolimbic dopaminergic projections, which are more limited to anterior (prefrontal) systems, may accentuate the precision of executive processing that underlies secondary consciousness; that is theory of mind, planning self-awareness and so on (Kilner et al., 2009; Noudoost and Moore, 2011). In summary, the dissociable effects of different modulatory neurotransmitters on conscious processing in the brain may be reducible to the anatomical specificity and the selective biasing of message passing in hierarchical infrastructures. Clearly, there is a wealth of psychopharmacological evidence that speaks to these issues, which we will consider in a subsequent paper. We conclude with some empirical predictions that follow from the theoretical arguments reviewed in this section.

## 6 Some empirical predictions

We now conclude by looking at some empirical predictions that emerge from our review of REM sleep and the PGO system in the light of generalized predictive coding and free energy minimization. There are several rather specific predictions about the functional anatomy of REM sleep that are informed by predictive coding architectures and the optimization of generative models that support consciousness. We will briefly consider predictions about the phenomenology, neuroanatomy, neurophysiology and neuropharmacology of REM sleep.

### 6.1 Dream phenomenology

If dream content is the brain's attempt to find plausible explanations for fictive visual searches of its environment, why is it is often so far from being veridical? If the brain's generative model is a near optimal model of the real world, why do dreams entertain scenarios that are so far from reality and pedestrian experience? From a functional perspective, to minimize the complexity of generative models it is necessary to explain (fictive) percepts in a parsimonious and as general a way as possible. Clearly, the generality of these explanations can only be established over a diverse range of dream content—otherwise the brain would fall into the trap of overfitting its internal model to a limited, and possibly idiosyncratic, repertoire of perceptual states. In other words, finding order in the real world may not be the same as finding order in the virtual world (Llewellyn, 2011). This suggests an optimal balance between rehearsing what has already been learned about the world and exploring new hypotheses and possibilities that could be experienced. This may, in part, explain the curious nature of dream content and be related to the creative and synthetic capacity of the brain that can be harnessed in wakefulness (Hobson and Wohl, 2005). Interestingly, the implicit neuromodulatory differentiation between waking and sleep consciousness may be impaired in neuropsychiatric conditions characterized by disorganization of the psyche (Llewellyn, 2011). It is perhaps worth noting, that the bizarreness of dream content is largely relational nature. In other words, it is the continuity and associations among percepts that appears to be violated; however, we only dream about things we could perceive, even if they could not exist in the context established the dream.

If REM sleep favors primary versus secondary aspects of consciousness, then dreaming should be characterized by a surplus of non-veridical predictions and a paucity of appropriate cognitive identifications (in relation to waking perception). We mean this in the sense that if there is cholinergic modulation of the precision of prediction errors in sensory hierarchies – that is not matched by an equivalent increase in the precision at higher (prefrontal) levels – cognitive and conceptual explanations will be driven primarily by fictive and unconstrained perceptual representations. This appears to be the case: Dream content is ‘bizarre’ because discontinuity and incongruity are entertained in the face of physical impossibilities. Dream percepts therefore cannot be reconciled with the waking percepts identified by the dreamer in generating his report (Hobson et al., 2000). This defective rationalization of dream phenomenology can be identified and measured by comparing it to reflective thought in waking (Hobson, 2011).

### 6.2 Neuroanatomy

One clear anatomical prediction is that there should be direct and reciprocal connections between the cortex and pontine nuclei. This is because cortical predictions about oculomotor proprioception should be delivered directly to the pontine nuclei and these connections should be reciprocated (with forward connections conveying prediction error). In short, although there are direct connections from the pontine system to the lateral geniculate (Calvo et al., 1992), there should also be direct connections to the cortical regions involved in the elaboration of PGO waves. A direct projection from pons to cortex has, in fact, been demonstrated (Laurent et al., 1977) and a cortico-pontine pathway can be inferred from experiments showing that REM sleep eye movements are ‘simplified’ following lesions of the visual cortex (Mouret et al., 1965).

### 6.3 Neurophysiology

In terms of electrophysiology, one would predict that the event related potentials measured with electroencephalography (or event related fields with magnetoencephalography) that are time locked to saccadic eye movements in wake and sleep are generated using the same system but with one important difference: the postsynaptic gain of superficial pyramidal cells (or more generally the principal cells elaborating forward-type connections) in the lateral geniculate body and primary visual cortex should be reduced. In other words, one local change in synaptic gain that is restricted to the early visual pathway (that we presume is mediated by a change in aminergic neuromodulation) should be sufficient to explain the distributed responses observed empirically during eye movements in sleep and wake. Recent developments in the modeling of electrophysiological responses now allow us to test this hypothesis using biophysically informed models of how electromagnetic signals are generated. This is called dynamic causal modeling (David et al., 2006) and allows one to compare different models of distributed electromagnetic signals that are generated by the PGO system. In this instance, one should be able to compare dynamic causal models of event related responses time-locked to the onset of saccadic eye movements (in waking and REM sleep).

For example, comparing models with and without direct and reciprocal connections between (hidden) subcortical pontine and cortical sources would constitute a test of the anatomical prediction above. There is already a literature on the use of dynamic causal modeling to make inferences about forward and backward connections (Garrido et al., 2007) and these techniques have been applied recently to look at backward connections from the frontal cortex in patients with impaired conscious level (Boly et al., 2011). Electrophysiological predictions about synaptic gain can be tested using dynamic causal models that allow for condition (wake versus sleep) specific changes in the postsynaptic gain of principal cells within specific sources (Kiebel et al., 2007). In this context, synaptic gain is modeled explicitly as a key component of intrinsic connectivity within sources of electromagnetic signals. The prediction here is that gain will increase during REM sleep in extrastriate and other cortical sources responsible for the generation of orienting saccadic eye movements (lateral occipital, parietal and frontal eye fields) but will decrease selectively in sources comprising the early visual pathways (lateral geniculate nuclei and striate cortex). Note that the nice thing about the PGO system, from the point of view of dynamic causal modeling, is that it is a very well-characterized system (Datta et al., 1998) in which the (hypothetical) source of proprioceptive prediction errors – driving geniculate and occipital responses – can be measured empirically from eye movements.

### 6.4 Neuropharmacology

Finally, one might anticipate that the same profile of changes in postsynaptic gain should be seen under pharmacological manipulations that emulate the aminergic suppression of sensory prediction errors. In other words, visually cued saccadic eye movements should come to resemble PGO waves, with pharmacological reductions in aminergic neurotransmission. In principle, this hypothesis can be tested using dynamic causal modeling in combination with pharmacological manipulations of noradrenergic and serotonergic neurotransmission. The technology to do this has been established using animal models and a series of developmental and pharmacological manipulations (Moran et al., 2008).

While this review has focused on animal models, the advent of brain imaging technology and advances in dynamic causal modeling now make it possible to study things like the PGO system in (un-anaesthetized) humans, while awake and asleep. The future of dream and consciousness science is thus bright. Data already in hand indicates that not only the visual system has predictive powers: other sensory systems exhibit it too (Hong et al., 2008), indicating that the idea of the brain as a model of the world is a theory whose time for scientific validation has come.

## 7 Conclusion

We have reviewed the empirical nature of sleep with a special focus on neuromodulation and the PGO system. We then considered sleep in terms of model optimization, under the free energy principle. The basic idea here is that the brain uses sleep to optimize its generative model of the world during wakefulness. This is akin to post hoc model selection, in which redundant parameters are removed to minimize model complexity and provide a more parsimonious internal model.

Whether model optimization of this sort is sufficient to explain why sleep is subject to evolutionary pressure is not an easy question to answer; however, this perspective provides a principled explanation for the utility of sleep that underwrites the homoeostatic and autopoietic imperatives for biological organisms. Furthermore, it becomes especially relevant for the complex brains that support sleep. Notably, the mechanisms of model optimization are exactly the same as those proposed by the AIM model: Namely, an inactivation of sensory afferents that rests upon input gating, which is mediated through modulatory neurotransmitter systems. It is interesting that the abstract treatment provided by the free energy approach and the empirically grounded AIM model converge on exactly the same conclusions.

Having said this, there are many issues that are unresolved by the brief theoretical treatment presented in this review. As noted above, associating synaptic plasticity and regression with model optimization does not speak to the established differences between slow wave and REM sleep at a neurophysiological or cognitive level. For example, the association between SWS and procedural memory and a more complicated relationship between REM and declarative memory suggests a regional specificity for putative synaptic optimization—a specificity that may be related to the distinction between hippocampal-dependent and independent consolidation (Ravassard et al., 2009; Ribeiro et al., 2002). We have focused on REM sleep and the phenomenology of PGO waves and dreaming. The mechanics of predictive coding provide a compelling three-way link between the physiology of PGO waves, rapid eye movements and the fictive percepts of dreaming. By association, this suggests that the sort of complexity minimization seen in REM sleep may pertain to systems involved in perceptual categorization (and declarative memory). Conversely, in SWS synaptic changes may conform to the same basic principles but in systems concerned more directly with motor control and procedural representations.

What is the basic explanation of sleep on offer here? As put nicely by one of our reviewers: “On the one hand, there is a requirement that the senses are shut down so complexity can be minimized. On the other hand, there is the idea that the senses are shut down because there is a learnt regularity about precision – namely that it drops at night – and that the brain simply continues free energy minimization under these conditions. Perhaps these ideas can be combined but, at least initially, they are different: the former says there is evolutionary pressure to sleep, the latter that sleep is a contingent upshot of the fact our free energy minimization happens on a planet that spins.”

The reviewer favored the latter explanation and we tend to agree: free energy minimization is all about making the brain a good model of its environment. This means that the brain – and indeed the phenotype – cannot be divorced from its environment. In this setting, natural selection can be regarded as selecting phenotypes (models) with the lowest free energy—or maximizing free fitness in evolutionary theory (Sella and Hirsh, 2005). This means that there is no necessary requirement to suppress sensory input to minimize free energy (or complexity); however, certain species have found a local optimum in a free fitness landscape (see Fig. 1d) that exploits night-time to focus on minimizing complexity. In this view, sleep is an opportunistic – and highly effective – process that allows the brain to concentrate on statistical housekeeping and can be regarded as an example of meta-selection—the selection of selective processes. In short, evolution has selected brains that sleep and sleep selects the synaptic connections that constitute brains, where both evolution and sleep minimize free energy or maximize free fitness.

Finally, we have discussed the implications of our theory for the study of wake and dream state phenomenology and made some specific predictions that can be tested with the modeling of electrophysiological responses associated with saccadic eye movements in waking and sleep. In future work, we hope to address these predictions using dynamic causal modeling of noninvasive electromagnetic recordings. We also hope to demonstrate the computational principles outlined in this paper using simulations of sleep based upon the same models of active inference we have used previously to simulate action observation.

## Figures and Tables

**Table 1:** Waking and REM sleep dreaming and consciousness: contrasting phenomenological, physiological and thermoregulatory factors.

	Waking	REM sleep
Phenomenology	Perception entrained by sensation	Perception sequestered from sensation
	Abstract concepts	Concrete concepts
	Orientation preserved	Orientation lost
	Emotions restrained	Emotions enhanced
	Memory intact	Memory for remote events unavailable

Behavior	Adaptive	Inactivated

Physiology	Brain activated	Brain activated
	Input–output gates open	Input–output gates closed
	Aminergic modulation	Cholinergic modulation

Homoeothermy	Intact (in mammals and birds with REM sleep)	Suspended (with closure of input–output gates)

Free energy	Free energy minimized:	Free energy minimized:
	by suppressing prediction errors—including interoceptive (thermoreceptor) errors	by suppressing the complexity of predictions—with no (thermoregulatory) adaptive responses

## Box 1 Free-energy

$$\begin{array}{ll} F(s,\mu ) & ={\text{D}}_{KL}(Q(\vartheta |\mu )||P(\vartheta |s))-\ln P_{a}(s|\eta )\\ \; & ={\text{D}}_{KL}(Q(\vartheta |\mu )||P(\vartheta |\eta ))-{\text{E}}_{Q}(\ln P_{a}(s|\vartheta )) \end{array}$$

*Free energy and perception*: Free energy is a function of a probability density function Q(ϑ|μ) that describes or approximates *posterior beliefs* about the causes ϑ(t) of sensory data s(t). These beliefs are encoded by *sufficient statistics* μ(t) that correspond to (changing) physical brain states. In the above equation, η denotes the form or structure of the brain's generative model that is needed to evaluate free energy. The first equality shows that free energy is always bigger than surprise $-\ln P_{a}(s|\eta )$, because the first (Kullback-Leibler divergence) term is always greater than zero. This term is the difference between the approximate and true posterior beliefs, such that when free energy is minimized, Q(ϑ|μ) becomes the true posterior P(ϑ|s) over the causes of observed sensory input. This corresponds to Bayes-optimal perceptual inference, under a generative model of the world (Helmholtz, 1866/1962; Barlow, 1974; Gregory, 1980; Dayan et al., 1995; Friston et al., 2006).

*Generative models and action*: To compute the free energy one needs the probability density functions $P_{a}(s|\vartheta )$ and P(ϑ|η). These are known as the *likelihood* and *priors* in statistics. Together, they comprise a *generative model* $P_{a}(s,\vartheta |\eta )=P_{a}(s|\vartheta )P(\vartheta |\eta )$ that expresses, probabilistically, how various causes in the world conspire to produce sensory data. These causes can be varied in nature; here we consider a (minimal) partition that distinguishes between states of the world that change quickly and causal regularities that change slowly. Furthermore, we make a distinction between states and their precision. The resulting three quantities are *states*, *precisions* and *parameters*: ϑ=(x,γ,θ), where precision corresponds to the inverse variance of random fluctuations of states. Posterior beliefs or expectations about these quantities are represented by physical brain states: $\mu =(\mu_{x},\mu_{\gamma },\mu_{\theta })$. Usually, expectations about states are thought to be encoded by *synaptic activity*, expectations about precision are encoded by *synaptic gain* and expectations about parameters are encoded by *synaptic efficacy* or connection strengths (see Fig. 3). Action, denoted by a(t), redeploys sensory epithelia and therefore determines sensory samples; this dependency is denoted by the subscript in the expression for the likelihood: $P_{a}(s|\vartheta )$.

## Box 2 Models and complexity

$$\begin{array}{ll} F(s,\mu ) & ={\text{D}}_{KL}(Q(\vartheta |\mu )||P(\vartheta |s))-\ln P_{a}(s|\eta )\\ \; & ={\text{D}}_{KL}(Q(\vartheta |\mu )||P(\vartheta |\eta ))-{\text{E}}_{Q}(\ln P_{a}(s|\vartheta )) \end{array}$$

The second equality above says that free energy is *complexity* plus *sensory surprise*. Sensory surprise is just the improbability of sensory samples, under posterior beliefs about how those samples were generated. The first (complexity) term is the divergence between *posterior beliefs* and *prior beliefs*: it is called complexity because it reports the degree to which prior beliefs have to be abandoned to predict sensory samples accurately and minimize sensory surprise. Complexity corresponds to the degrees of freedom or number of parameters that are called upon to explain data. A good model has low complexity and uses a small number of parameters to provide a parsimonious explanation for observed data (cf., Occam's razor). One can unpack the complexity term as follows:

$${\text{D}}_{KL}(Q(\vartheta )||P(\vartheta ))={\text{D}}_{KL}(Q(x,\gamma )||{\text{E}}_{Q(\theta )}P(x,\gamma |\theta ))+{\text{D}}_{KL}(Q(\theta )||P(\theta |\eta ))$$

This decomposes complexity into a complexity over time varying states (and their precision) and a complexity over parameters. Crucially, prior beliefs about states are controlled by posterior beliefs about parameters; while prior beliefs about the parameters P(θ|η) are controlled by quantities η that can be regarded as the presence or absence of a synaptic connection. The key point here is that complexity can be suppressed by changing prior beliefs (technically, these can be empirical priors that depend upon model parameters or full priors that depend on the model per se; these are the first and second terms above). Intuitively, this means one can improve models by removing redundant parameters to optimize prior beliefs. In our context, this corresponds to pruning redundant synaptic connections.
